# MnCo_2_O_4_/NiCo_2_O_4_/rGO as a Catalyst Based on Binary Transition Metal Oxide for the Methanol Oxidation Reaction

**DOI:** 10.3390/nano12224072

**Published:** 2022-11-18

**Authors:** Mohammad Bagher Askari, Sadegh Azizi, Mohammad Taghi Tourchi Moghadam, Majid Seifi, Seyed Mohammad Rozati, Antonio Di Bartolomeo

**Affiliations:** 1 Department of Semiconductor, Institute of Science and High Technology and Environmental Sciences, Graduate University of Advanced Technology, Kerman 7631818356, Iran; 2 Department of Physics, Faculty of Science, University of Guilan, Rasht 413351914, Iran; 3 Department of Physics “E. R. Caianiello”, University of Salerno, 84084 Fisciano, SA, Italy

**Keywords:** reduced graphene oxide, methanol electrooxidation, MnCo_2_O_4_/NiCo_2_O_4_/rGO, methanol fuel cell

## Abstract

The demands for alternative energy have led researchers to find effective electrocatalysts in fuel cells and increase the efficiency of existing materials. This study presents new nanocatalysts based on two binary transition metal oxides (BTMOs) and their hybrid with reduced graphene oxide for methanol oxidation. Characterization of the introduced three-component composite, including cobalt manganese oxide (MnCo_2_O_4_), nickel cobalt oxide (NiCo_2_O_4_), and reduced graphene oxide (rGO) in the form of MnCo_2_O_4_/NiCo_2_O_4_/rGO (MNR), was investigated by X-ray diffraction (XRD), scanning electron microscope (SEM), and energy-dispersive X-ray (EDX) analyses. The alcohol oxidation capability of MnCo_2_O_4_/NiCo_2_O_4_ (MN) and MNR was evaluated in the methanol oxidation reaction (MOR) process. The crucial role of rGO in improving the electrocatalytic properties of catalysts stems from its large active surface area and high electrical conductivity. The alcohol oxidation tests of MN and MNR showed an adequate ability to oxidize methanol. The better performance of MNR was due to the synergistic effect of MnCo_2_O_4_/NiCo_2_O_4_ and rGO. MN and MNR nanocatalysts, with a maximum current density of 14.58 and 24.76 mA/cm^2^ and overvoltage of 0.6 and 0.58 V, as well as cyclic stability of 98.3% and 99.7% (at optimal methanol concentration/scan rate of 20 mV/S), respectively, can be promising and inexpensive options in the field of efficient nanocatalysts for use in methanol fuel cell anodes.

## 1. Introduction

Due to the high dependence of the modern world on electricity, the energy supply is a major concern for many countries. The severe reduction in fossil fuel sources and the harmful effects of excessive use of these fuels on the environment has led the world to use clean and renewable fuel sources. Energy storage and energy production from these renewable sources are two important topics that have attracted the attention of many researchers in various fields [1,2].

Supercapacitors and electrochemical batteries as energy storage equipment [3,4] and solar cells, wind turbines, and fuel cells as energy production–conversion types of equipment [5] have attracted the attention of researchers [6]. Among fuel cells, alcoholic fuel cells have specific properties such as lower operating temperature [7], ease of use, and portability [8]. Moreover, in alcoholic fuel cells, the problems caused by the storage and conversion of hydrogen in fuel cells have been solved. Therefore, due to these characteristics, alcoholic fuel cells have been considered by researchers more than other fuel cells [9].

In a direct methanol fuel cell, methanol oxidation and oxygen reduction occur at the anode and cathode [10], and a Nafion is placed between the anode and cathode as a membrane capable of proton exchange [11]. Among alcohol fuel cells, methanol fuel cells have become popular due to the simple structure of methanol as a fuel in them [12].

The best and most efficient catalysts for the MOR process in alcoholic fuel cells are platinum-based catalysts and other rare and expensive metals [13,14,15]. Considering that these materials are unrivaled and have attracted the attention of researchers in the field of methanol oxidation [16], their high cost is an obstacle to the industrialization of these catalysts. Studies have shown that doping [17], hybridizing [18], and combining [19] these materials with low-cost materials such as metal–organic frameworks (MOFs) [20], types of carbon [21,22,23], or other materials [24,25,26,27,28] is an attractive way to introduce cost-effective catalysts [29]. However, the introduction of a catalyst with no presence of rare metals can still be a bright prospect for researchers in the field of materials and catalysts. Among the proposed materials as catalysts for alcohol oxidation, sulfides and oxidized metals are considered more than other materials [30]. The simple synthesis of these materials, the low cost of their precursors compared to platinum, and their excellent cyclic stability are their advantages over platinum-based composites. Although none of the proposed materials in the alcohol oxidation process can compete with platinum, they can be promising options for advancing energy production from low-cost materials. Typical materials proposed as catalysts for alcoholic fuel cells are Zn_1−x_Mn_x_Co_2_O_4_/rGO [31], Co_3_O_4_-Ni_3_S_4_-rGO [32], Fe_3_O_4_@MoS_2_/RGO [33], PSS/MnO_2_/rGO [34], Ni/NiO/MWCNT [35], NiCo_2_O_4_/rGO [36], RuO_2_-MnCo_2_O_4_/rGO [37], MoS_2_/Ni_3_S_2_/rGO [38], and ZnO-MWCNT @ Fe_3_O_4_ [39]. Most researchers believe that the main disadvantage of metal oxides and sulfides for use as catalysts is their low electrical conductivity [40].

To increase the contact between reactants and the catalyst, the structure of the electrode must be porous, and the high porosity provides a large, involved surface area with reactants, which allows them to reach active sites easily [41].

Metal oxides, in many cases, do not have a significant active site on their surfaces; however, the active surface can be controlled by various synthesis methods [42]. Furthermore, the main challenge related to low electrical conductivity can be solved by composting these materials with conductive materials or doping conductive metals in their structure [43].

One of the attractive options for introducing low-cost catalysts is applying a variety of carbonaceous materials, including carbon nanotubes, reduced graphene oxide, or biomass carbon in the structure of metal oxide-based catalysts. Thus, by using carbon as the most abundant and available element in nature, we have improved the electrical conductivity of catalysts [44,45].

In this study, MN and MNR nanocatalysts coated on nickel foam (NF) were synthesized by a one-step hydrothermal method to investigate their methanol oxidation ability. Considering the fact that few studies have been conducted to investigate the capability of two BTMOs as catalysts in the MOR process, the main novelty and importance of our research is to introduce a new catalyst that includes two BTMOs as well as an easy synthesis process. The role of rGO, as a promoter component in the oxidation process, was considered, and the presented results show that rGO plays an important role in improving the catalyst performance in the methanol oxidation process.

## 2. Materials and Methods

All precursors used in this study, including manganese nitrate, cobalt nitrate, nickel nitrate, urea, potassium hydroxide, and methanol, were purchased with purity of more than 99% from Merck. X-ray diffraction analysis was performed by EQUINOX INEL3000, and SEM imaging was provided by Phenom Prox. Potentiostat/Galvanostat AUTOLAB3202N device and related electrode systems were used for the electrochemical studies.

### 2.1. Synthesis and Characterization

#### 2.1.1. Synthesis of MnCo on a Nickel Foam Substrate

Firstly, nickel foam was washed through the three-time sonication process with 0.5 M hydrochloric acid and deionized water (DI) for 10 min. Then, 234 mg of Mn (NO_3_) 2 with 464 mg of Co (NO_3_)_2_⋅6H_2_O, 150 mg of urea, and 28 mg of NH_4_F in a solution containing 60 mL of DI water and 10 mL of ethanol were stirred and dissolved for 30 min. Finally, the cleaned nickel foam (1 × 1 cm^2^) was placed in an autoclave containing the prepared solution and maintained for 12 h at 140 °C. The autoclave cooled, and the electrode was washed several times with DI water and ethanol and dried for 4 h at 45 °C.

#### 2.1.2. Synthesis of MnCo_2_O_4_/NiCo_2_O_4_

To synthesize MnCo_2_O4/NiCo_2_O_4_, 3 mmol Co (NO_3_)_2_⋅6H_2_O with 1.5 mmol Ni (NO_3_)_2_⋅6H_2_O and 18 mmol urea were dissolved in a solution with 30 mL of distilled water and 20 mL of pure ethanol and stirred for 45 min. Then, the nickel foam coated with MnCo was placed in an autoclave containing the solution at 145 °C for 11 h. Finally, after washing and drying at 40 °C, the electrode was annealed at 400 °C.

#### 2.1.3. Synthesis of MnCo_2_O_4_/NiCo_2_O_4_/rGO

To synthesize MnCo_2_O_4_/NiCo_2_O_4_/rGO, 3 mg of rGO was prepared by the Hummers method and was added gradually to the solution that was previously prepared for MnCo_2_O_4_/NiCo_2_O_4_.

## 3. Results and Discussion

### 3.1. Characterizations

**Figure 1:**
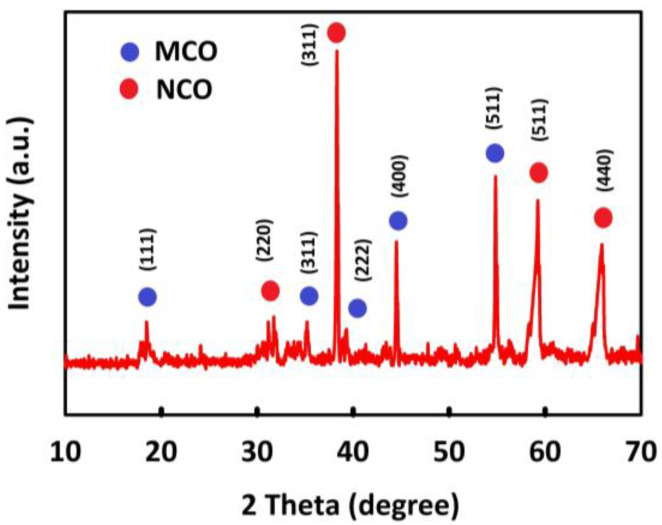
XRD patterns of MnCo_2_O_4_/NiCo_2_O_4_.

To investigate the crystal structure of the synthesized nanomaterials, XRD analysis was performed using a 3000 EQUINOX INEL device with a CuKα lamp at room temperature. The corresponding peaks were observed at angles 2θ from 10° to 80°. Figure 1 shows the XRD pattern for MnCo_2_O_4_/NiCo_2_O_4_. As can be seen, the index peaks at 18.3°, 36.1°, 37.8°, 43.6°, and 57.9° correspond to the Miller indices (111), (311), (222), (400), and (511), respectively, in agreement with the reference data (JCPDS card: 23-1237) [46], which confirms the spinel structure of MnCo_2_O_4_; moreover, the index peaks 31.3°, 36.8°, 59.2°, and 64.9° confirm the presence of NiCo_2_O_4_ spinel with the Miller indices (220), (311), (511), and (440), conforming to the reference data (JCPDS card 20-0781) [47]. The NiCo_2_O_4_ index peaks are visible at some angles of reflection with the MnCo_2_O_4_ index peaks, so the XRD results confirm the presence of both NiCo_2_O_4_ and MnCo_2_O_4_ spinel.

**Figure 2:**
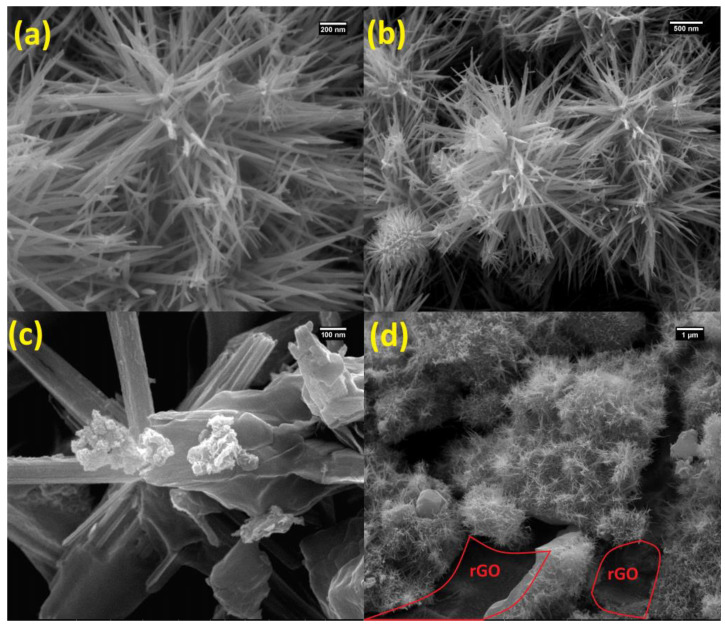
SEM images of MN (**a**,**b**) and MNR (**c**,**d**).

SEM analysis was used to investigate the surface morphology of the MN and MNR nanocomposites. For this purpose, SEM images were prepared using a SEM microscope device (Phenom Prox, Waltham, MA, USA) at different magnifications. Figure 2a,b shows the MnCo_2_O_4_/NiCo_2_O_4_ SEM images at different scales. Regarding the SEM images, the obtained morphology may provide the appropriate contacting surface between the catalyst and methanol, which can be a positive factor to facilitate the methanol oxidation process. Considering the MNR, Figure 2c,d also clearly shows the MnCo_2_O_4_/NiCo_2_O_4_ hybrid placed on the surface of rGO nanosheets. The uniform dispersion of the MN catalyst on the surface of the rGO is also clearly visible, and rGO has been marked in Figure 2d.

**Figure 3:**
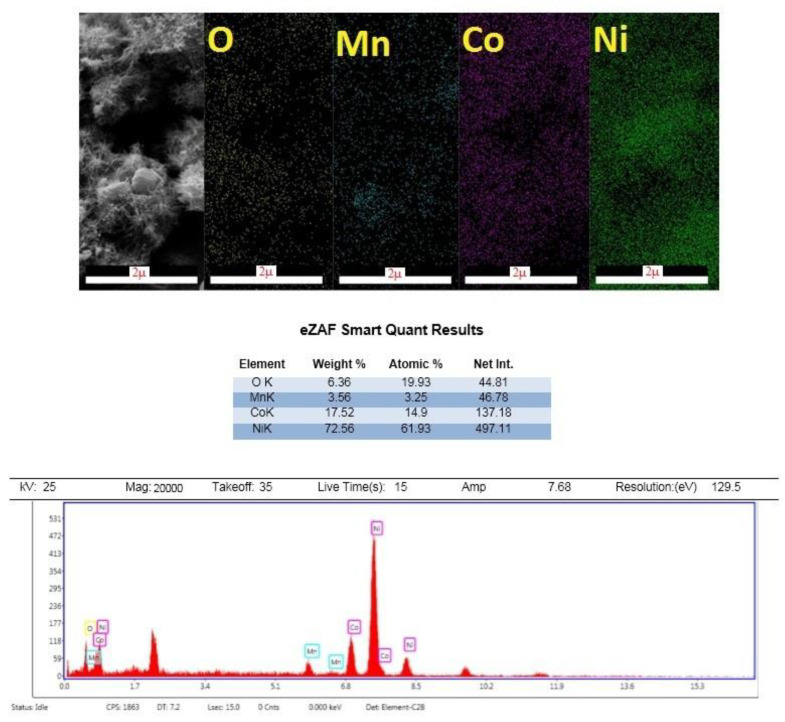
EDX mapping analysis of MnCo_2_O_4_/NiCo_2_O_4_.

The EDX mapping analysis is an appropriate way to investigate the presence of manganese, nickel, cobalt, and oxygen. In this system, the number of X-rays emitted is counted against their energy. The amount of energy of each X-ray determines the characteristic of the element from which the beam is emitted. Therefore, by obtaining the energy spectrum against the counted X-rays, the quantitative and qualitative estimates of the elements in the sample are examined. Figure 3 also shows the EDX diagram, which confirms the presence of the elements Mn, Co, O, and Ni on the nickel foam substrate.

### 3.2. Electrochemical Studies

#### 3.2.1. Electrode Preparation

The electrochemical properties of the catalysts were studied on a Potentiostat/Galvanostat (AUTOLAB3202N, Wuhan, China) device with a typical three-electrode set-up, involving MnCo_2_O_4_/NiCo_2_O_4_ and MnCo_2_O_4_/NiCo_2_O_4_/rGO coated on NF as working electrodes, Ag/AgCl as the reference electrode, and 0.5 mm diameter platinum wire as the auxiliary electrode.

#### 3.2.2. Electrochemical Investigation of Catalysts for Methanol Oxidation

According to many studies, metal oxide-based nanocatalysts are more efficient in the oxidation process of alcohols in alkaline media. Therefore, we investigated the capability of MN and MNR nanocatalysts in the methanol oxidation process. In the initial part, the cyclic voltammetric analysis of the nanocatalysts was performed in a 2 M KOH solution at a scan rate of 20 mV/s.

**Figure 4:**
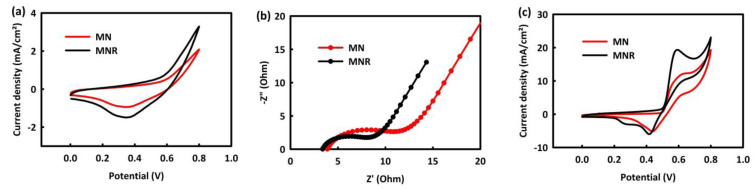
CV curves of MN and MNR (**a**), EIS plots of MN and MNR in 2M KOH (**b**), and CV curves of the MN and MNR 2 M KOH and in the presence of 0.5 M methanol (**c**).

As can be seen in Figure 4a, both nanocatalysts have capacitive behavior, in that these materials are used as supercapacitor electrodes for electrochemical energy storage devices. Based on the CV analysis of catalysts in KOH, the MNR catalyst has a higher current density than the MN catalyst. The electrochemical impedance spectroscopy (EIS) analysis was performed to further study the electrical properties of catalysts. According to Figure 4b, the EIS analysis was accomplished at open circuit potential (OCP) over the frequency range of 0.01 Hz–100 kHz in a 2 M KOH aqueous solution. The charge transfer resistance (RCT) for MN and MNR in an alkaline medium obtained 9 and 6 ohms, respectively.

The ability of catalysts for MOR was evaluated by performing CV analysis in 2 M KOH solution in the presence of 0.5 M methanol in the potential range of 0 to 0.8 mV.

Figure 4c shows that both nanocatalysts have a methanol oxidation peak in their CV analysis, which confirms the efficiency of the catalysts in the oxidation of methanol. It can also be seen that the oxidation peaks observed in MNR are much higher than in MN. Two important points can be concluded from Figure 4a–c: both nanocatalysts have the ability for MOR and can be used in the anode of methanol fuel cells, and the performance of MNR is better than MN in the methanol oxidation reaction. The MNR nanocatalyst has lower charge transfer resistance than MN and has a higher oxidation current density. This advantage can be related to the presence of rGO in the structure of the catalyst, which improved the performance of the catalyst by increasing the electrical conductivity [48] as well as by increasing the electrochemically active surface area [49,50,51]. In the following, we examine the influence of various parameters such as methanol concentration, scan rates, and temperature in MOR by MNR and MN, and we optimize these parameters for two nanocatalysts.

##### The Effect of Methanol Concentration on MOR Process by MN and MNR

To study the effect of methanol concentration on the MOR process of MN and MNR nanocatalysts, the CV analysis was performed in the constant concentration of 2 M KOH with different concentrations of methanol (0.3, 0.5, 0.7, 1, 1.5, 2, 2.5, and 3 M) at a scan rate of 20 mV/s.

**Figure 5:**
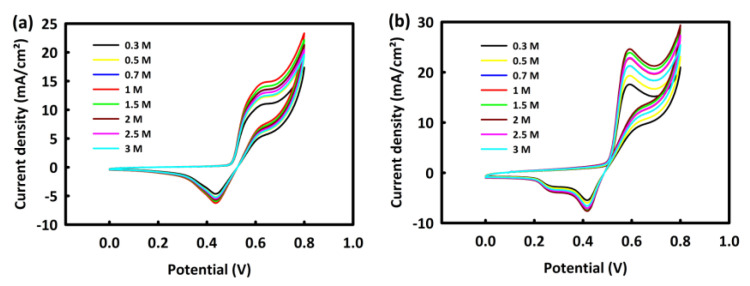
CV curves of MN (**a**) and MNR (**b**) at different concentrations of methanol.

According to Figure 5a, for the MN catalyst, the oxidation peak has an upward behavior up to 1 M methanol, and then at 1.5 and 2 M methanol concentrations, the peak oxidation current density decreases. In the same behavior, the MNR catalyst showed an upward trend in methanol oxidation activity up to 2 M methanol, and then from 2.5 M methanol, the oxidation current density trend decreased (Figure 5b). It appears that at concentrations higher than the critical concentration of methanol, the surface of the catalyst is saturated and the methanol oxidation process is disrupted.

##### Investigation of the Effect of Scan Rates on MOR Process by MN and MNR

**Figure 6:**
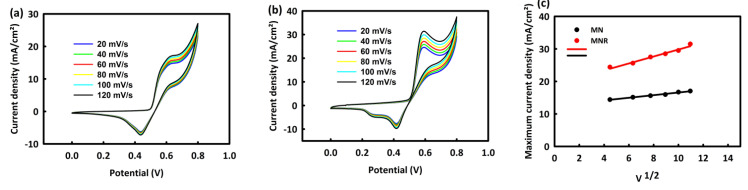
CV curves of MN (**a**) and MNR (**b**) at different scan rates and (**c**) plot of methanol concentration versus maximum current density.

By choosing one and two molar concentrations of methanol as the optimal concentrations for the two nanocatalysts, MN and MNR, in the process of MOR at different scan rates (20, 40, 60, 80, 100, and 120 mV/s), we investigate the behavior of nanocatalysts by increasing the scan rate. Figure 6a,b shows the CV analysis of MN and MNR at an optimal concentration of methanol in the presence of a 2 M alkaline solution of KOH at different scan rates. As seen in the behavior of both nanocatalysts, capacitive and faradic currents increase with the increasing scan rate. The square root of the scan rate is plotted in terms of the maximum current density for two catalysts in Figure 3c. The linear relationship between these two parameters, with R^2^ = 0.998 and 0.997 for the two catalysts, indicates the diffusion control mechanism in the methanol oxidation process.

The proposed mechanism of methanol oxidation by nanocatalysts can be proposed in the form of a six-electron mechanism, as follows:

$$\mathrm{Catalyst}\;+{\mathrm{CH}}_{3}\mathrm{OH}\to \mathrm{Catalyst}\;-{\mathrm{CH}}_{3}{\mathrm{OH}}_{\mathrm{ads}}$$

$$\mathrm{Catalyst}\;-{\mathrm{CH}}_{3}{\mathrm{OH}}_{\mathrm{ads}}+4\mathrm{OH}¯\to \mathrm{Catalyst}\;-{\left(\mathrm{CO}\right)}_{\mathrm{ads}}+4H_{2}O+4e¯$$

$$\mathrm{Catalyst}\;+\mathrm{OH}¯\to \mathrm{Catalyst}\;-{\mathrm{OH}}_{\mathrm{ads}}+\;e¯$$

$$\mathrm{Catalyst}\;-{\mathrm{CO}}_{\mathrm{ads}}+\mathrm{Catalyst}\;-{\mathrm{OH}}_{\mathrm{ads}\;}+\mathrm{OH}¯\;\to \mathrm{Catalyst}\;+{\mathrm{CO}}_{2}+H_{2}O+e¯$$

##### Investigation of the Effect of Temperature on the MOR Process by MN and MNR

**Figure 7:**
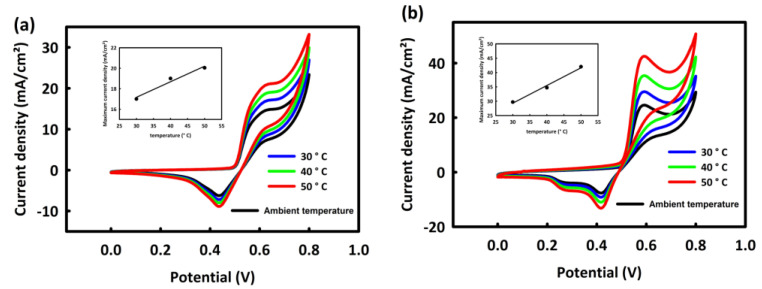
CV curves of MN (**a**) and MNR (**b**) at different temperatures.

As mentioned in the introduction, one of the most important features of methanol fuel cells is the performance of these cells at relatively lower temperatures than other fuel cells. So, to investigate the behavior of MN and MNR catalysts at various temperatures, a series of CV analyses were performed at optimal concentrations and scan rates of 20 mV/s for two nanocatalysts at different temperatures (ambient temperature, 30, 40, and 50 °C). It can be seen from Figure 7a,b that the oxidation current increases with increasing peak temperature. This increase in the current density can be related to the increase in temperature to facilitate OH^−^ adsorption by the catalyst [38]. Moreover, the rising temperature can affect the activation energy of reactants and provide sufficient energy to break chemical bonds of the methanol in the methanol oxidation reaction. Therefore, it can be said that with increasing temperature, the oxidation process of methanol is facilitated by two nanocatalysts. The linear relationship between the temperature rise and maximum oxidation peak current density is shown in the inset of Figure 7a,b.

##### MN and MNR Stability Evaluation in MOR Process at Optimal Scan Rate and Concentration

**Figure 8:**
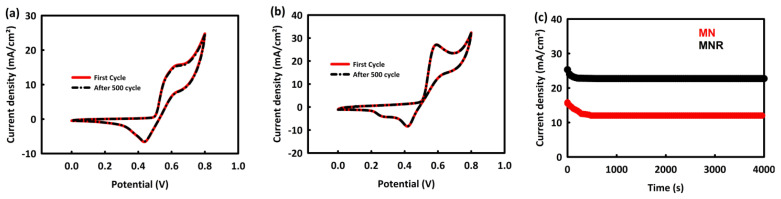
Cyclic stability of MN (**a**) and MNR (**b**) in MOR process after 500 consecutive CV and (**c**) chronoamperometry analyses of MN and MNR.

To evaluate the stability of nanocatalysts in the methanol oxidation process, 500 consecutive CV cycles of MN and MNR were performed in an alkaline solution at an optimal concentration and scan rate of 60 mV/s. As can be seen, the MNR is almost 99.7% stable after this number of cycles and there is no decrease in its oxidation current density peak (Figure 8a), and this stability rate for MN is about 98.3% (Figure 8b). In order to check the stability of the synthesized nanocatalysts in the MOR process, chronoamperometry analysis was performed at a potential of 0.6 V for a duration of 4000 s. As seen in Figure 8c, MNR and MN have 89.8% and 75.9% stability in current density, respectively. Although both nanocatalysts have adequate stability in the MOR process, the superiority of MNR over MN can also be related to its higher electrical conductivity and higher electrochemical active surface area, which is due to the presence of rGO in the structure of the nanocatalyst.

The performance of the MNR catalyst in the process of methanol oxidation is compared with other reported studies in Table 1. Examining Table 1 shows that the proposed catalyst is competitive with other research in terms of overvoltage and current density.

## 4. Conclusions

In this study, we introduced nanocatalysts based on transition metal oxides. In this regard, we synthesized the MN and MNR nanocatalysts deposited on the surface of NF by a simple hydrothermal method. After the physical characterization of the nanocatalysts, we studied the capability of nanocatalysts in the MOR process. Although both nanocatalysts performed relatively well in methanol oxidation, the MNR catalyst is more efficient in the MOR process due to the presence of rGO as a two-dimensional material with suitable conductivity and an active surface area. The two proposed nanocatalysts, MN and MNR, with a current density of 14.58 and 24.76 mA/cm^2^ and 98.3% and 99.7% cyclic stability, respectively, after 500 consecutive CV cycles in the MOR process, can be inexpensive and attractive candidates for use in the anode of methanol fuel cells.

## Figures and Tables

**Table 1:** Comparison of electrochemical properties of MNR in MOR with some reported works.

Electrode Material	Current Collector	Methanol Concentration (M)	Scan Rate (mV s^−1^)	Anodic Potential (V)	Peak Current Density (mA cm^−2^)	Ref.
NiCo_2_O_4_/rGo	GCE	0.1 M KOH/0.5 M Methanol	50	0.256	16.6	[52]
NiO-MOF/rGO	GCE	1 M NaOH/3 M Methanol	50	0.8	275.85	[53]
α-CoMoO_4_ nanoflakes	Carbon cloth	1 M KOH/0.5 M Methanol	50	0.8	25	[54]
NiCo_2_O_4_/carbon xerogel	GCE	0.5 M NaOH/0.5 M Methanol	50	0.29	98	[55]
FeO/NiO MOF	GCE	1 M NaOH/3 M Methanol	50	0.85	486.14	[56]
NiMoO_4_/C	Carbon paper	1 M KOH/2 M Methanol	50	0.45	49	[57]
Zn1−xMnxCo2O4/rGO with x = 0.4	GCE	1 M KOH/0.5 M Methanol	50	0.6	142.3	[58]
Mn_3_O_4_-Co_3_O_4_-rGO	GCE	1 M KOH/1 M Methanol	100	0.48	16.5	[59]
Mn_3_O_4_-CeO_2_-rGO	GCE	1 M KOH/0.8 M Methanol	90	0.51	17.7	[1]
MNR	Nickel Foam	2 M KOH/2 M Methanol	20	0.58	24.76	This work

## Data Availability

Data are available on request from the authors.

## References

[1] Askari M.B., Rozati S.M., Di Bartolomeo A. (2022). Fabrication of Mn_3_O_4_-CeO_2_-rGO as Nanocatalyst for Electro-Oxidation of Methanol. Nanomaterials.

[2] Razmjoo A., Kaigutha L.G., Rad M.V., Marzband M., Davarpanah A., Denai M. (2021). A Technical analysis investigating energy sustainability utilizing reliable renewable energy sources to reduce CO_2_ emissions in a high potential area. Renew. Energy.

[3] Feng S., Chen J., Ma L., Wu J., Lin J., Liao J., Lu X., Yan X., Zeng S., Xi Y. (2022). Hierarchical nanoarchitecture of vanadium disulfide decorated 3D porous carbon skeleton with improved electrochemical performance toward Li ion battery and supercapacitor. Ceram. Int..

[4] Mehtab T., Yasin G., Arif M., Shakeel M., Korai R.M., Nadeem M., Muhammad N., Lu X. (2019). Metal-organic frameworks for energy storage devices: Batteries and supercapacitors. J. Energy Storage.

[5] Shaygan M., Ehyaei M., Ahmadi A., Assad M.E.H., Silveira J.L. (2019). Energy, exergy, advanced exergy and economic analyses of hybrid polymer electrolyte membrane (PEM) fuel cell and photovoltaic cells to produce hydrogen and electricity. J. Clean. Prod..

[6] Askari M.B., Salarizadeh P. (2020). Binary nickel ferrite oxide (NiFe_2_O_4_) nanoparticles coated on reduced graphene oxide as stable and high-performance asymmetric supercapacitor electrode material. Int. J. Hydrog. Energy.

[7] Siwal S.S., Thakur S., Zhang Q., Thakur V.K. (2019). Electrocatalysts for electrooxidation of direct alcohol fuel cell: Chemistry and applications. Mater. Today Chem..

[8] Ozoemena K.I., Musa S., Modise R., Ipadeola A.K., Gaolatlhe L., Peteni S., Kabongo G. (2018). Fuel cell-based breath-alcohol sensors: Innovation-hungry old electrochemistry. Curr. Opin. Electrochem..

[9] Askari M.B., Salarizadeh P., Beheshti-Marnani A., Di Bartolomeo A. (2021). NiO-Co3O4-rGO as an Efficient Electrode Material for Supercapacitors and Direct Alcoholic Fuel Cells. Adv. Mater. Interfaces.

[10] Liu F., Yang X., Dang D., Tian X. (2019). Engineering of hierarchical and three-dimensional architectures constructed by titanium nitride nanowire assemblies for efficient electrocatalysis. ChemElectroChem.

[11] Wang P., Cui H., Wang C. (2022). Ultrathin PtMo-CeOx hybrid nanowire assemblies as high-performance multifunctional catalysts for methanol oxidation, oxygen reduction and hydrogen oxidation. Chem. Eng. J..

[12] De Sá M., Pinto A., Oliveira V. (2022). Passive direct methanol fuel cells as a sustainable alternative to batteries in hearing aid devices–An overview. Int. J. Hydrog. Energy.

[13] Chen A., Holt-Hindle P. (2010). Platinum-based nanostructured materials: Synthesis, properties, and applications. Chem. Rev..

[14] Jiang Y., Guo Y., Zhou Y., Deng S., Hou L., Niu Y., Jiao T. (2020). Synergism of Multicomponent Catalysis: One-Dimensional Pt-Rh-Pd Nanochain Catalysts for Efficient Methanol Oxidation. ACS Omega.

[15] Zuo Y., Sheng W., Tao W., Li Z. (2022). Direct methanol fuel cells system: A review of dual-role electrocatalysts for oxygen reduction and methanol oxidation. J. Mater. Sci. Technol..

[16] Tian H., Wu D., Li J., Luo J., Jia C., Liu Z., Huang W., Chen Q., Shim C.M., Deng P. (2022). Rational design ternary platinum based electrocatalysts for effective methanol oxidation reaction. J. Energy Chem..

[17] Yuan G., Wang L., Zhang X., Wang Q. (2019). Self-supported Pt nanoflakes-doped amorphous Ni (OH) 2 on Ni foam composite electrode for efficient and stable methanol oxidation. J. Colloid Interface Sci..

[18] Tao L., Shi Y., Huang Y.C., Chen R., Zhang Y., Huo J., Zou Y., Yu G., Luo J., Dong C.L. (2018). Interface engineering of Pt and CeO2 nanorods with unique interaction for methanol oxidation. Nano Energy.

[19] Zhang X., Ma J., Yan R., Cheng W., Zheng J., Jin B. (2021). Pt-Ru/polyaniline/carbon nanotube composites with three-layer tubular structure for efficient methanol oxidation. J. Alloy. Compd..

[20] Ma L., Gan M., Ding J., Han S., Wei D., Shen J., Zhou C. (2020). MOF-derived N-doped carbon coated CoP/carbon nanotube Pt-based catalyst for efficient methanol oxidation. Int. J. Hydrog. Energy.

[21] Bhuvanendran N., Ravichandran S., Zhang W., Ma Q., Xu Q., Khotseng L., Su H. (2020). Highly efficient methanol oxidation on durable PtxIr/MWCNT catalysts for direct methanol fuel cell applications. Int. J. Hydrog. Energy.

[22] Wang H., Yang Y., Ren Y., Chen D., Wei J., Wang L., Xie A., Luo S. (2021). Electrochemical synthesis of Pt nanoparticles on ZrO2/MWCNTs hybrid with high electrocatalytic performance for methanol oxidation. J. Electroanal. Chem..

[23] Yang C., Jiang Q., Li W., He H., Yang L., Lu Z., Huang H. (2019). Ultrafine Pt nanoparticle-decorated 3D hybrid architectures built from reduced graphene oxide and MXene nanosheets for methanol oxidation. Chem. Mater..

[24] Liu Y., Hu B., Wu S., Wang M., Zhang Z., Cui B., He L., Du M. (2019). Hierarchical nanocomposite electrocatalyst of bimetallic zeolitic imidazolate framework and MoS2 sheets for non-Pt methanol oxidation and water splitting. Appl. Catal. B Environ..

[25] Niu W., Li L., Liu X., Zhou W., Li W., Lu J., Chen S. (2015). One-pot synthesis of graphene/carbon nanospheres/graphene sandwich supported Pt3Ni nanoparticles with enhanced electrocatalytic activity in methanol oxidation. Int. J. Hydrog. Energy.

[26] Su S., Zhang C., Yuwen L., Liu X., Wang L., Fan C., Wang L. (2016). Uniform Au@ Pt core–shell nanodendrites supported on molybdenum disulfide nanosheets for the methanol oxidation reaction. Nanoscale.

[27] Tang B., Lv Y., Du J., Dai Y., Pan S., Xie Y., Zou J. (2019). MoS_2_-coated Ni_3_S_2_ nanorods with exposed {110} high-index facets as excellent CO-tolerant cocatalysts for Pt: Ultradurable catalytic activity for methanol oxidation. ACS Sustain. Chem. Eng..

[28] Zhou Q., Pan Z., Wu D., Hu G., Wu S., Chen C., Lin L., Lin Y. (2019). Pt-CeO_2_/TiN NTs derived from metal organic frameworks as high-performance electrocatalyst for methanol electrooxidation. Int. J. Hydrog. Energy.

[29] Yu F., Xie Y., Tang H., Yang N., Meng X., Wang X., Tian X.L., Yang X. (2018). Platinum decorated hierarchical porous structures composed of ultrathin titanium nitride nanoflakes for efficient methanol oxidation reaction. Electrochim. Acta.

[30] Askari M.B., Salarizadeh P., Di Bartolomeo A., Beitollahi H., Tajik S. (2021). Hierarchical nanostructures of MgCo_2_O_4_ on reduced graphene oxide as a high-performance catalyst for methanol electro-oxidation. Ceram. Int..

[31] Li Z., Li B., Chen J., Pang Q., Shen P. (2019). Spinel NiCo2O4 3-D nanoflowers supported on graphene nanosheets as efficient electrocatalyst for oxygen evolution reaction. Int. J. Hydrog. Energy.

[32] Askari M.B., Rozati S.M. (2022). Construction of Co_3_O_4_-Ni_3_S_4_-rGO ternary hybrid as an efficient nanoelectrocatalyst for methanol and ethanol oxidation in alkaline media. J. Alloy. Compd..

[33] Askari M.B., Beheshti-Marnani A., Seifi M., Rozati S.M., Salarizadeh P. (2019). Fe3O4@ MoS2/RGO as an effective nano-electrocatalyst toward electrochemical hydrogen evolution reaction and methanol oxidation in two settings for fuel cell application. J. Colloid Interface Sci..

[34] Baruah B., Kumar A. (2018). PEDOT: PSS/MnO_2_/rGO ternary nanocomposite based anode catalyst for enhanced electrocatalytic activity of methanol oxidation for direct methanol fuel cell. Synth. Met..

[35] Askari M.B., Salarizadeh P., Seifi M., Rozati S.M. (2019). Ni/NiO coated on multi-walled carbon nanotubes as a promising electrode for methanol electro-oxidation reaction in direct methanol fuel cell. Solid State Sci..

[36] Wei Z., Guo J., Qu M., Guo Z., Zhang H. (2020). Honeycombed-like nanosheet array composite NiCo_2_O_4_/rGO for efficient methanol electrooxidation and supercapacitors. Electrochim. Acta.

[37] Askari M.B., Rozati S.M., Salarizadeh P., Saeidfirozeh H., Di Bartolomeo A. (2021). A remarkable three-component RuO_2_-MnCo_2_O_4_/rGO nanocatalyst towards methanol electrooxidation. Int. J. Hydrog. Energy.

[38] Salarizadeh P., Askari M.B., Di Bartolomeo A. (2022). MoS_2_/Ni_3_S_2_/Reduced graphene oxide nanostructure as an electrocatalyst for alcohol fuel cells. ACS Appl. Nano Mater..

[39] Moghadam M.T.T., Seifi M., Askari M.B., Azizi S. (2022). ZnO-MWCNT@Fe_3_O_4_ as a novel catalyst for methanol and ethanol oxidation. J. Phys. Chem. Solids.

[40] Ling T., Zhang T., Ge B., Han L., Zheng L., Lin F., Xu Z., Hu W.B., Du X.W., Davey K. (2019). Well-dispersed nickel-and zinc-tailored electronic structure of a transition metal oxide for highly active alkaline hydrogen evolution reaction. Adv. Mater..

[41] Li Z., Ye L., Lei F., Wang Y., Xu S., Lin S. (2016). Enhanced electro-photo synergistic catalysis of Pt (Pd)/ZnO/graphene composite for methanol oxidation under visible light irradiation. Electrochim. Acta.

[42] Zaman S., Huang L., Douka A.I., Yang H., You B., Xia B.Y. (2021). Oxygen reduction electrocatalysts toward practical fuel cells: Progress and perspectives. Angew. Chem..

[43] Tariq I., Asghar M.A., Ali A., Badshah A., Abbas S.M., Iqbal W., Zubair M., Haider A., Zaman S. (2022). Surface Reconstruction of Cobalt-Based Polyoxometalate and CNT Fiber Composite for Efficient Oxygen Evolution Reaction. Catalysts.

[44] Li Z., Xu S., Xie Y., Wang Y., Lin S. (2018). Promotional effects of trace Bi on its highly catalytic activity for methanol oxidation of hollow Pt/graphene catalyst. Electrochim. Acta.

[45] Zaman S., Su Y.Q., Dong C.L., Qi R., Huang L., Qin Y., Huang Y.C., Li F.M., You B., Guoet W. (2022). Scalable Molten Salt Synthesis of Platinum Alloys Planted in Metal–Nitrogen–Graphene for Efficient Oxygen Reduction. Angew. Chem..

[46] Kong X., Zhu T., Cheng F., Zhu M., Cao X., Liang S., Cao G., Pan A. (2018). Uniform MnCo_2_O_4_ porous dumbbells for lithium-ion batteries and oxygen evolution reactions. ACS Appl. Mater. Interfaces.

[47] Han B., Song J., Liang S., Chen W., Deng H., Ou X., Xu Y.J., Lin Z. (2020). Hierarchical NiCo_2_O_4_ hollow nanocages for photoreduction of diluted CO2: Adsorption and active sites engineering. Appl. Catal. B Environ..

[48] Agrawal P.R., Kumar R., Teotia S., Kumari S., Mondal D., Dhakate S.R. (2019). Lightweight, high electrical and thermal conducting carbon-rGO composites foam for superior electromagnetic interference shielding. Compos. Part B Eng..

[49] Jeevitha G., Abhinayaa R., Mangalaraj D., Ponpandian N., Meena P., Mounasamy V., Madanagurusamy S. (2019). Porous reduced graphene oxide (rGO)/WO_3_ nanocomposites for the enhanced detection of NH 3 at room temperature. Nanoscale Adv..

[50] Mehta S.S., Nadargi D.Y., Tamboli M.S., Alshahrani T., Minnam Reddy V.R., Kim E.S., Mulla I.S., Park C., Suryavanshi S.S. (2021). RGO/WO_3_ hierarchical architectures for improved H2S sensing and highly efficient solar-driving photo-degradation of RhB dye. Sci. Rep..

[51] Sekar K., Raji G., Tong L., Zhu Y., Liu S., Xing R. (2020). Boosting the electrochemical performance of MoS_2_ nanospheres-N-doped-GQDs-rGO three-dimensional nanostructure for energy storage and conversion applications. Appl. Surf. Sci..

[52] Umeshbabu E., Rao G.R. (2016). NiCo2O4 hexagonal nanoplates anchored on reduced graphene oxide sheets with enhanced electrocatalytic activity and stability for methanol and water oxidation. Electrochim. Acta.

[53] Noor T., Zaman N., Nasir H., Iqbal N., Hussain Z. (2019). Electro catalytic study of NiO-MOF/rGO composites for methanol oxidation reaction. Electrochim. Acta.

[54] Padmanathan N., Shao H., Selladurai S., Glynn C., O’Dwyer C., Razeeb K.M. (2015). Pseudocapacitance of α-CoMoO4 nanoflakes in non-aqueous electrolyte and its bi-functional electro catalytic activity for methanol oxidation. Int. J. Hydrog. Energy.

[55] El-Deeb M.M., El Rouby W.M., Abdelwahab A., Farghali A.A. (2018). Effect of pore geometry on the electrocatalytic performance of nickel cobaltite/carbon xerogel nanocomposite for methanol oxidation. Electrochim. Acta.

[56] Noor T., Mohtashim M., Iqbal N., Naqvi S.R., Zaman N., Rasheed L., Yousuf M. (2021). Graphene based FeO/NiO MOF composites for methanol oxidation reaction. J. Electroanal. Chem..

[57] Jothi P.R., Kannan S., Velayutham G. (2015). Enhanced methanol electro-oxidation over in-situ carbon and graphene supported one dimensional NiMoO4 nanorods. J. Power Sources.

[58] Rebekah A., Anantharaj S., Viswanthan C., Ponpandian N. (2020). Zn-substituted MnCo2O4 nanostructure anchored over rGO for boosting the electrocatalytic performance towards methanol oxidation and oxygen evolution reaction (OER). Int. J. Hydrog. Energy.

[59] Askari N., Askari M.B., Di Bartolomeo A. (2022). Electrochemical Alcohol Oxidation and Biological Properties of Mn3O4-Co3O4-rGO. J. Electrochem. Soc..

